# What level of competency do experienced nurses expect from a newly graduated registered nurse? Results of an Australian modified Delphi study

**DOI:** 10.1186/s12912-016-0166-2

**Published:** 2016-07-22

**Authors:** Roy A. Brown, Patrick A. Crookes

**Affiliations:** School of Nursing, Faculty of Science, Medicine and Health, University of Wollongong, Wollongong, New South Wales Australia

**Keywords:** Competence, Nursing, New graduate RNs, Nursing education

## Abstract

**Background:**

Individuals who have recently completed accredited courses and are eligible to register as a nurse in Australia are often referred to as not being ‘work-ready’ by clinically based colleagues. This project identified the level of competence that can be reasonably expected of a newly registered nurse (RN) graduating in Australia. The research was undertaken using the necessary skills identified by Crookes and Brown in 2010.

**Methods:**

A consensus methodology using a modified Delphi technique invited experienced nurses to identify the level of competency expected by the new RN in each of the skills areas.

**Results:**

More than half of respondents did not believe that new graduates could practice independently in 18 of the 30 skills areas. There were only four skills areas where more than two thirds of the respondents believed the new graduate could operate independently.

**Conclusions:**

There is a lack of clarity about the level of competency of the newly graduating registered nurse in Australia. The profession and employers need clarity regarding the areas and level of competence that can reasonably be expected of a newly graduated RN. Utilising the findings of this research will enable the skills and competencies to be integrated into eligibility to practice programmes. Further research needs to be undertaken to review the foci of nursing preparation programmes to meet the needs of novice practitioners and the health care consumer population.

**Electronic supplementary material:**

The online version of this article (doi:10.1186/s12912-016-0166-2) contains supplementary material, which is available to authorized users.

## Background

The aim of this paper is to present the findings from using a modified Delphi technique which explored the professions expectations of the newly graduated RN’s competence using the 30 skills areas identified by Crookes and Brown in 2010 [1] (Additional file 1). Respondents were invited to identify the level of competence of a new graduate RN in Australia at the point of registration using Bondy’s [2] criteria. These criteria were used as two thirds of Australian universities used the Bondy criteria [2] to assess their nursing students as they progressed through their eligibility to practice programmes (Crookes and Brown) [1].

This paper is one aspect of a larger multisite study conducted across multiple Australian universities and health care providers. The larger project was funded by the Australian Learning and Teaching Council (ALTC) and supported by the Council of Deans of Nursing and Midwifery - Australia and New Zealand (CDNM-ANZ). This paper focuses specifically on the nursing professions views of the competency levels of a newly graduating RN. Other aspects of the larger project are reported elsewhere (Authors own).

### Literature regarding the competency level of a newly registered nurse

A literature search to locate and review research on competency levels for newly graduated RNs explored a range of databases: the details of which can be seen in Additional file 2.

The papers although not entirely focussed on the new graduates ranged between 2003 and 2014. The main themes will be explored briefly in this section; key themes centred on ‘feeling competent vs feeling incompetent’; ‘skills related to specific client groups’ and ‘what skills are taught and practiced as opposed to what are needed and why’.

In the first theme “feeling competent on graduation” a range of clinical skills or procedures were identified in which new graduates did not ‘feel’ competent to perform (Adair et al. [3]; Dlamini et al. [4]; Liou et al. [5]; Liou and Cheng [6]); both new graduates and senior nursing clinicians supported this perception. This led to the exploration of ‘core’ and ‘advanced’ skills and competency highlighted by Liou et al. [5] however this lacks clarity and requires further research. Students express the need for more practice within their programmes of study, even in the UK where the students spend 2300 h in a variety of settings – they still lack confidence (Bradshaw and Merriman [7], Farrand et al. [8], Ross & Clifford [9], Dolan [10]). Duchscher [11] explored ‘transition shock’; the notion of ‘professional adjustment’ and the ‘feelings of anxiety, insecurity and inadequacy’ which manifest as a lack of competence and/or capability The majority of research focussed on acute tertiary care settings and so has limited value for a comprehensively prepared graduate RN.

In the second theme, “skills related to specific client groups” new graduate skills were identified by a number of authors in order to meet the needs of ‘the most common clinical presentations’. Burns and Poster [12] identified ’10 high risk, high volume’ conditions with nurse executives in order to explore the skills and competencies that new graduates would need to care for this client group. Patterson et al. [13] identified the creation of orientation programmes in emergency nursing for new graduates which centred on ‘classes organized by body system or diagnosis’. and ‘emergency nursing skills were incorporated into the program’. These evaluated well but participants were still concerned about skill acquisition and practice and so competence. Lastly in relating skills development to specific client groups Patterson et al. [14] explored Mental Health Nursing; four main themes were identified and then 14 competencies however these significantly overlapped with the NMBA [15] competencies. There were a number of competencies that were broadly stated but may well be viewed as more ‘specific’ to mental health nursing such as ‘protection from aggression’ and ‘managing unsafe behaviour’.

“What skills are taught and practiced as opposed to what skills are needed and why”: Brown et al. [16] undertook an audit of skills taught in nursing eligibility to practice programmes so that a documentary analysis could be undertaken. From this analysis, and a number of modified Delphi rounds, the thirty skills areas were derived. These thirty skills areas were then circulated to nurses (academics, clinicians and managers *n* = 495) across Australia to identify whether the skills taught in nursing eligibility to practice programmes were ’necessary’ for a new graduate RN. This work illustrated that there were a great many skills taught but following the modified Delphi rounds the thirty skills areas were identified by respondents as ‘applicable’ and ‘necessary’ for a new graduate RN. Expert nurses involved in the preparation of new graduates were asked what a new RN needed to be competent in practice (Birks et al. [17]). This showed a disparity between what was taught and what was actually needed – again a somewhat acute care focus was noted.

In summary much of the identified literature has a number of foci; firstly there is on the whole a focus on the new graduate in an acute care hospital setting, and secondly self-report was the main means of identifying the new graduates’ competence. There were some variations however Burns and Poster [12] did also include an expert nurse’s assessment of the new graduates’ competence in acute care practice. Patterson et al. [14] considered Mental health practice and Crookes and Brown [1] surveyed a range of clinicians and academics from differing clinical backgrounds. This literature and these strategies provide limited acute care focused evidence regarding the newly graduated RN’s skills set, relatively small numbers were used and self-report provides limited evidence.

The intention of this research, in the light of the literature, was to consider the skill set required of a comprehensively prepared RN through the gaze of experienced registered nurses, clinicians, managers, researchers and academics. Acknowledging the work of Brown et al. [16] in identifying and then verifying with nurses across Australia the thirty skills areas as being ‘necessary’ afforded a high degree of reliability that the skills areas were appropriate for a new graduate RN in Australia.

The competence of new RN graduates, both at the point of joining the workforce on graduation and as they gain experience, is an important dimension of quality and safety. Thus each nursing school and prospective employer has a vested interest in ensuring that the initial skills and competency of the new graduate and the conditions for the transition and the ongoing development of the new graduate RN are optimised.

Unlike in some countries, where there are conjoint and/or national accreditation systems in place (NMC [18]) national accreditation was not introduced in Australia until 2010. Prior to this each state and territory had local accreditation processes in place. This pre-2010 variation within the Australian sectors validation processes probably contributed to a lack of homogeneity in terms of what one might expect of a newly graduating RN.

The sheer geographical size of Australia probably affects local course delivery and expectation of new graduate RN capabilities and competence. Universities are located across states and territories with twenty two metropolitan and seventeen rural or remote; this compounds the variability in the development and the delivery of nursing programmes; and subsequently the competency and skills of the new RN. This when combined with the differing interpretation and application of the NMBA [15] competency statements across eligibility to practice programmes in Australia led to a variation in the competence and skill set of the newly graduating registered nurse. Interestingly this is not solely an Australian phenomenon; Burns and Poster [12] identified, through a discussion with senior nurses and deans and heads of schools in Texas that nurses from different programmes in Texas, USA performed better than others from different schools within the same state. This very fluid space concerning what skills might be taught and assessed; what level of competence was expected and what competency assessment tools were being used prompted the initial, timely ALTC study.

## Method

### Aim

This paper is drawn from a larger project which sought to develop a competency assessment schedule for use in eligibility to practice programmes in Australia Crookes & Brown [1]. The main aim of this paper is to identify the competency level of newly registered nurses in the ‘necessary’ skills areas (Brown et al.) [16] as perceived by experienced RNs.

### Design of the study

An inclusive, consensus methodology using a modified Delphi technique was employed to maximise contributions and ownership from the profession. The use of modified Delphi rounds enables experts in their field (in this case experienced nurses and academics) to be engaged in refining the levels of competence expected in a new graduate nurse (Delbecq, Van de Ven & Gustafson [19]). A modified Delphi technique allows each respondent to contribute equally. No respondents are aware of other participant’s responses at the point when they complete the survey – thus minimising any possible power differentials influencing respondent’s rankings; so reducing bias. Any number of iterations or cycles can be used until only a few or no minor changes are suggested by respondents. At the end of each of the modified Delphi rounds there was a section for respondents to make suggestions or to add comments.

The 30 skills areas (Crookes and Brown [1]) utilised were circulated to respondents in the modified Delphi survey to identify the competency levels. The level of competency was measured using Bondy’s [2] modified criteria because of its widespread use in Australian nursing schools (Table 1). Two measures of central tendency were used; “mean” and “mode” in order to give the reader both an ‘average’ view of the data and the highest number of respondents for each of the thirty skills areas. The work is specifically exploring the respondent’s views about whether the new graduate is independent/competent therefore descriptive percentages are used. Numerical values were assigned to the Bondy [2] categories to facilitate this analysis (Table 1); were “independent” was attributed 5 and “dependent” was attributed 1.

**Table 1:** Bondy [2] Modified criteria used to classify students competence

Independent: (I)	5	Refers to being safe & knowledgeable; proficient & coordinated and appropriately confident and timely. Does not require supporting cues.
Supervised: (S)	4	Refers to being safe & knowledgeable; efficient & coordinated; displays some confidence and undertakes activities within a reasonably timely manner. Requires occasional supporting cues.
Assisted: (A)	3	Refers to being safe and knowledgeable most of the time; skilful in parts however is inefficient with some skill areas; takes longer than would be expected to complete the task. Requires frequent verbal and some physical cues.
Marginal: (M)	2	Refers to being safe when closely supervised and supported; unskilled and inefficient; uses excess energy and takes a prolonged time period. Continuous verbal and physical cues.
Dependent: (D)	1	Refers to concerns about being unsafe and being unable to demonstrate behaviour or articulate intention; lacking in confidence, coordination and efficiency. Continuous verbal and physical cues/interventions necessary.

This paper reports specifically on the expected competency level of the new graduate at the point of registration. Other aspects of this round and findings of subsequent rounds of the survey are reported elsewhere (Authors own).

### Participants

A number of strategies were used to recruit respondents for an earlier survey that was part of the larger project. Heads/Deans of nursing schools delivering RN programmes in Australia were obtained through the CDNM-ANZ, these universities were invited to participate and to identify an individual within their school to act as a liaison with the researcher (*n* = 39). These individuals then assisted in identifying local participants for the modified Delphi rounds. Additionally members of the Australian Nursing and Midwifery Council Professional Reference Group (ANMC PRG) and the Chief Nursing Officers in each state and territory were asked to circulate the information through their contacts to reach as many members of the profession as possible. These combined strategies led to a respondent population of nearly 500 participants from a range of nursing backgrounds. Respondents were contacted by email with a link to the survey.

### Data collection

An on line survey tool was used (Survey Gizmo) to host the survey and manage the data. The Bondy [2] criteria to be used were identified and were accessible on each page of the online survey to assist the respondents in completing the survey (Table 1). The survey tool comprised of the 30 skills items and open ended questions inviting respondents to add possible missing skills areas or competencies. The presentation of the questions within the survey was rotated in order to avoid respondent fatigue (Oppenheim [20]); this is a recognised technique to reduce such bias. A three week time frame was established so that respondents had sufficient time to complete the survey.

The feedback from the modified Delphi technique was reviewed and then re-presented for verification with the respondents, at this point 100 respondents were randomly invited to review the list of thirty skills areas both in terms of and content and ranking and 89 responded confirming that this ranking was accurate and reflected their expectation.

### Data analysis

Following completion of the survey, all data were downloaded as Excel and SPSS files for analysis (Statistical Package for the Social Sciences Version 19). The raw data was reviewed for completeness and missing data. Interestingly there was very little missing data; only a few responses were not answered by some respondent’s time in role and area of practice contained the most missing data. There were no specific items that were consistently missed. Two measures of central tendency were calculated; the mean and mode score for each skill area.

Free text comments were analysed thematically by two independent reviewers. The main findings from this aspect of the survey are reported in a later paper.

Finally the expert panel members (Project Management Group, ANMC-PRG and Chief Nurses Group) were presented with the findings and discussed the analysis and final scores and rankings and agreed the rationale for the final list of skills areas seen in Additional file 1.

## Results and discussion

### Demographics of the respondents

Initially 495 nurses were invited to participate through using the sampling frame and Survey Gizmo, of those 299 (60.4 %) responded; 27.3 % were academics (*n* = 80); 68.7 % (*n* = 203) worked within clinical settings and 4 % (*n* = 12) of respondents were no longer currently working in nursing (Additional file 3). Of the respondents who worked in practice, senior clinicians totalled 45 %; a number of these were in education roles Clinical Nurse Educators (21 %) and Graduate Programme Coordinators (5 %). A number of new graduates on transition programmes contributed to the survey (2 %). The remaining population consisted of Directors of Nursing Services or their assistants/deputies (9 %). The nurse academics who participated were an experienced group, with 78 % (*n* = 66) Lecturer or Senior Lecturers; 17 % (*n* = 13) Professors or Associate Professors and the remainder Deans/Heads of Nursing Schools.

A range of clinical specialties were represented by respondents; there were a significant number (*n* = 91; 38.2 %) who were involved in more than one clinical setting, the highest proportion of these were managers, clinical nurses and educators. Just over a quarter (*n* = 61; 25.5 %) respondents were employed in acute care tertiary settings such as medicine, surgery, critical care and perioperative practice. Fewer respondents were employed in aged care (*n* = 30; 12.1 %), primary care (*n* = 14; 5.6 %) and mental health (*n* = 43; 17.26 %). Nearly half of the respondents had been in their current roles in nursing from 3 to 10 years (*n* = 102; 42.5 %), with fewer respondents employed in their current role less than 2 years (*n* = 77; 32.08 %) or over ten years (*n* = 71; 29.58 %). These figures reflect the make-up of the nursing profession in Australia (AHPRA-NMBA [21]). The population also reflects the working clinical population with regard to clinical settings also strengthening the data.

### Delphi survey responses

For each of the 30 skills areas identified by Brown et al. [16], respondents were asked to identify the competency level they would expect of a newly graduating RN on completion of their eligibility to practice programme. If the respondent expected the new RN to be ‘competent’, in Bondy’s terms ‘Independent’ (scoring 5); this meant that the new graduate would ‘*be safe & knowledgeable; proficient & coordinated and appropriately confident and timely; that the new graduate would not require supporting cues’*. The ranked skills areas can be seen in Table 2. The top eight skills areas were identified as having a mean score of above 4.5; these are the skills areas that respondents expect new graduate RNs to be ‘work ready’.

**Table 2:** The ranked skills areas. [NB: the slight variations in the numbers are due to a small number of respondents who stated ‘not applicable’ or did not answer the question]

Skill Area Year3 (*n* = 299)	No. of respondents stating the new graduate would be independent (n)	Weighted Score	Percentage independent
Privacy and Dignity	244	4.81	83.849
Demonstrates behaviour conducive to learning	221	4.7	76.207
Personal care - ability to assess, plan implement and evaluate care of clients across a range of settings using a holistic, comprehensive nursing model	201	4.64	68.601
Efficient and Effective Communication	192	4.61	66.667
Communication and Documentation	183	4.56	63.322
Professional Nursing Behaviours - includes collaborative approaches to care	184	4.53	63.230
Therapeutic Nursing Behaviours/ Respectful of personal space	176	4.56	61.754
Preventing Risk and Promoting safety - Duty of care	179	4.53	61.301
Cultural Competence	164	4.47	56.747
Clinical monitoring and management - Use of assessment tools	153	4.45	52.577
Promoting self care	150	4.43	51.903
Critical Analysis & Reflective Thinking	149	4.42	51.557
Planning of Nursing Care	142	4.40	48.797
Learner/Evidence Based Practitioner	132	4.34	45.675
Coordinating Skills Regarding Nursing Process -uses a range of appropriate assessment strategies and skills across a range of settings	124	4.24	43.056
Teamwork and Multidisciplinary Team working	118	4.26	40.830
Learning and developmental culture - Learning environment	116	4.27	40.418
Clinical interventions - preparing, assisting after care (Investigations/surgery/diagnostic)	102	4.24	35.052
Medications and IV Products	101	4.22	34.708
Knowledge of key nursing implications of common medical/surgical patient presentations	95	4.12	32.872
Technology and Informatics	91	4.15	31.597
Different roles of RNs in different treatment or care settings	84	4.10	28.866
Demonstrates Teaching/Educator skills	69	3.86	24.211
Dementia related skills	69	3.97	23.875
Acts as a Resource	65	3.87	22.648
Dealing with emotional and bereaved people	61	3.89	21.034
Mental Health Nursing Care	57	3.86	19.655
Supervisory Skills	42	3.73	14.894
Leadership Skills	36	3.68	12.721
Case Manager	18	3.50	6.475

The skills area achieving the highest score was ‘Privacy and Dignity’; this was rated by 244 (83.8 %) of respondents as independent, followed by ‘Demonstrates behaviour conducive to learning’ with 221 (76.2 %) respondents. A number of respondents commented that several of the skills areas were “almost intuitive” and “crucially important in a new grad”. Some respondents suggested that students “should come to nursing” already implicitly understanding privacy and dignity. The idea of ‘approachability’ and ‘a supportive demeanour’ as well as ‘professional behaviour’ were noted. Only the top four (in Table 2) achieved more than 2/3’s of respondents expecting the new graduate RN to be independent.

Respondents believed that new graduate RNs are less competent in the three lowest ranked skills areas, three skills areas scored below 3.8 (where 3 is assisted and 4 is supervised), these were case manager (3.51), leadership (3.68) and supervisory skills (3.73). Only 6.5 % of respondents (*n* = 18) believed that a new graduate RN was competent to ‘case manage’. Interestingly 12 respondents stated that case manager was ‘not applicable’ for a new graduate RN.

In order to explore this data further a number of examples are analysed here to illustrate the results, the examples chosen are ranked across the range; the tenth ranked Clinical Monitoring and Management; nineteenth (Medications and IV Products) and thirtieth (Case Manager) see Table 2.

#### Clinical monitoring and management - use of assessment tools

For this skills area 52.58 % of respondents (*n* = 153) identified that a graduating RN would be independent and 40.8 % (*n* = 119) that they would expect them to require supervision. Two respondents stated that this skills area was ‘not applicable’ and 5.8 % believed the new graduate RN would require assistance. This finding supports Birks et al’s [17] who reported that the new graduate RN believed that they were taught assessment skills that they did not use, or used very rarely. With over half the respondents expecting that the new graduate RN would be competent this is an area of practice that requires further exploration.

#### Medications and IV products

An extremely important skills area in clinical practice; this item (ranked nineteen out of thirty in terms of the expected level of competence 4.22) 101 respondents (34.71 %) believed that the new graduate RN would be competent. Interestingly in terms of this being a ‘necessary skill area’, more than 98 % responded to the initial survey (Crookes and Brown) [1] stating that this was a necessary skill area for a newly graduated RN. More than 55 % of respondents (*n* = 160) believed that the new graduate RN would require some level of supervision. Supervision was defined as the new graduate RN being safe & knowledgeable; efficient & coordinated; displaying some confidence and undertakes this activity within a reasonable timely manner, but that they required the occasional supporting cue. Comments from the modified Delphi for this item illustrated how challenging the experienced nurses viewed how ‘near qualifying students/new RNs’ could be ‘independent’ in this skills area. Hence the mean for this skills area being closer to ‘supervised’.

Burns and Posters [12] reflected these findings concerning how competent graduating RNs self-reported their ‘feeling of competence’ with a number of medication administration examples. The issue concerning whether or not all new graduate RNs should be independent is an interesting debate; but an important area to explore as there is clearly a difference of ‘professional opinion’ across Australia, and the international literature (Burns and Poster [12]. Within this study a number of respondents believed that it ‘was not possible’ for a final year nursing student to be independent, whereas others explored how the student can be seen to be independent. Many explained using techniques such as ‘think out aloud’ (Lewis) [22] so that the assessor could be assured that the final year nursing student could clearly articulate what they were going to do, and more importantly, why they were going to undertake a particular form of assessment/monitoring and then why they would then escalate that assessment if necessary.

#### Case manager

This was the lowest ranked of the thirty skills areas (Table 2); the weighted score was 3.51 indicating that there is an expectation that the new graduate would have some grasp of the skill, but that on the whole the newly graduated registered nurse would be safe and knowledgeable most of the time; they would be skilful in parts however there would be some inefficiencies in some aspects of the skill area; they would take longer than would be expected to complete the task and they would probably require frequent verbal and some physical cues. Here only 18 respondents (6.48 %) believed that the new graduate RN would be independent and 146 (52.5 %) supervised. There is limited research in this area however it is a skill that both new graduate nurses and experienced nurses say that new grads are unable to perform (Burns & Poster [12], Adair et al. [3], Duchscher [11]).

The final section of the results and discussion section briefly explores the homogeneity of the responses regarding the different groups and clinical backgrounds of the respondents. The table in Additional file 4 illustrates three sub group examples but there is far too much detail to explore the full data set here. The table compares the mean scores within the whole population (299) and then acute care (29.2 %), academics (27 %) and mental health (13.7 %) respondents. The data presented in this way illustrates that the broadly homogeneous results indicate the level of agreement across nurses from different areas of nursing practice.

There was broad agreement between respondents regarding which skills areas newly graduating RN’s need to be able to perform in practice. ‘Privacy and Dignity’ ranked highest across all groups, ‘Demonstrates behaviour conducive to learning’ was ranked second or third by respondents. ‘Personal Care’ was ranked in the top five; ‘Efficient and effective communication’ ranked 3 or 4 across all respondents and ‘Communication and documentation’ are highly ranked. The lowest ranked skills areas were ‘Case manager’ with mean scores ranging from 3.37 to 3.69; ‘Leadership’ ranged from 3.61 to 3.71, and ‘Supervisory Skills. Also rated lowest consistently by all groups were ‘Dealing with emotional and bereaved people’, ‘Acts as a resource’ and ‘Mental Health nursing skills’ (apart from the Mental Health respondents were analysis of results identified a mean score 4.12 which for that subgroup placed this item slightly higher than the rest at 22^nd^ out of the 30 skills areas – the others placed this as 25^th^.

The relatively homogeneous data illustrates that the nursing profession in Australia broadly agree on what skills areas new graduates should be competent in and broadly what level of competence the profession, and more importantly, the key stakeholders expect of newly graduated registered nurse in those skills areas. This was confirmed in the second modified Delphi round in which there were no significant differences noted in the rankings by respondents.

The rankings presented in Table 2 and Additional file 4 illustrate the range and complexity of nursing practice and the difficulty when one is considering the level of competency that one might expect of a new graduate registered nurse at the point of graduation in Australia. The data within this study does illustrate that even though there is a tendency, using the combined score (the mean), in which 84 % believe the new graduate RN would be independent, there are 16 % who believe the new graduate is not competent. Views do differ across the nursing profession within Australia on what level of competence one might expect of a new graduate but there is broad agreement for each particular skill area. Newly registered nurses in Australia are ‘deemed competent’ on graduation in terms of the NMBA [15] National Competency Standards for the Registered Nurse.

The range in this study varies from 6.5 to 84 % of respondents believing that the new graduate should be independent, and therefore competent, in a given skills area (Table 2). For the ‘top four’ ranked skills areas (Table 2) more than 2/3 of respondents believed the new graduate would be competent. In the majority of skills areas however, more than three quarters, less than half the respondents believed that the newly graduated RN in Australia should be independent in those skills areas (Table 2). In the bottom four skills areas (Table 2) less than 20 % of respondents believed that the new graduate would be competent; this has significance on how new nursing graduate RNs are prepared.

## Conclusion

The study sought to identify the level of competency in the thirty skills areas (Brown et al. [16]) as perceived by registered nurses from a range of practice areas within Australia. The findings indicate that there is no clear expectation within the nursing profession that newly graduated RNs would be competent. This agrees with the literature that the new graduate RN is ‘not work ready’. The new graduate RN does not ‘feel ready’; this is supported by the profession in that the new graduate RN is not independent in at least 26 of the thirty skills areas.

This work has identified the level of competency that the nursing profession could reasonably expect of the newly graduating registered nurse against an agreed list of skills areas. Future work in this area could usefully focus on how those skills can be further developed in undergraduate preparation programmes in a range of clinical settings. This would include specialty areas of practice so there is continuity of skill and competency development. This could lead to two critical reviews; firstly a curriculum review of nursing programmes to examine competency and skill development and secondly consider the transition of newly graduating RNs in to practice and the type and duration of support required. Utilising the agreed skills areas and levels of competency within accreditation processes should reduce the dissonance about the apparent lack of competency of the newly graduating RN in Australia.

## Abbreviations

ALTC, Australian Learning and Teaching Council; ANMAC, Australian Nursing and Midwifery Accreditation Council; ANMC, Australian Nursing and Midwifery Council; CDNM, Council of Deans of Nursing and Midwifery; I.V. or I.V.I., intravenous infusion; MMSE, mini mental state examination; NMBA, Nursing and Midwifery Board of Australia; OLT, office of teaching and learning; RN, registered nurse; RUDAS, Rowland Universal Dementia Assessment Scale

## Additional files

Additional file 1: Thirty Skills areas from Crookes and Brown [1]; Brown et al. [16]. (DOCX 40 kb) Additional file 2: Literature Search Strategy. (DOCX 39 kb) Additional file 3: Participant demographics. (DOCX 40 kb) Additional file 4: Top and bottom six skills areas ranked presented by respondent role. (DOCX 41 kb)
