# Supplementary material for: What level of competency do experienced nurses expect from a newly graduated registered nurse? Results of an Australian modified Delphi study
Source: BMC Nurs. 2016 Jul 22;15:45. doi: 10.1186/s12912-016-0166-2 (PMC4957913; doi:10.1186/s12912-016-0166-2)
Supplement: Additional file 1: — Thirty Skills areas from Crookes and Brown [1]; Brown et al. [16]. (DOCX 40 kb) [file 12912_2016_166_MOESM1_ESM.docx]

Additional file 1: Thirty Skills areas from Crookes and Brown 2010 [1]; Brown et al 2015 [16].

1. Planning of Nursing Care (e.g. Range of varied settings/client’s needs)
2. Understanding the different roles of RNs in different treatment or care settings (e.g. Aged care, rural and remote, acute, mental health, etc.)
3. Medications and IV Products (e.g. Safe and appropriate administration of medications)
4. Clinical monitoring and management - Use of assessment tools (e.g. Hemodynamic/respiratory assessment, MMSE, RUDAS etc.) All forms of assessment are included here.
5. Technology and Informatics (e.g. IVI management systems, Patient Information Systems, etc.)
6. Personal care - ability to assess, plan implement and evaluate care of clients across a range of settings using a holistic, comprehensive nursing model
7. Mental Health Nursing Care (e.g. Application of assessment tools and care strategies and interventions)
8. Knowledge of key nursing implications of common medical/surgical patient presentations
9. Clinical interventions - Preparing, Assisting After care (Investigations/surgery/diagnostic)
10. Professional Nursing Behaviours - includes collaborative approaches to care (e.g. Advocacy, scope of practice, being aware of one’s self, etc.)
11. Privacy and Dignity (e.g. culturally acceptable practice, personal space, respectful)
12. Dealing with emotional and bereaved people (e.g. breaking bad news, dealing with anger, etc.)
13. Dementia related skills (e.g. Behavioural and Psychosocial Symptoms of Dementia)
14. Coordinating Skills Regarding Nursing Process -uses a range of appropriate assessment strategies and skills across a range of settings
15. Leadership Skills
16. Preventing Risk and Promoting safety - Duty of care (e.g. Strategies for reducing risk, risk assessment, etc.)
17. Case Manager (e.g. Coordination of care, crisis/emergency situation management, etc.)
18. Teamwork and Multidisciplinary Team working
19. Supervisory Skills
20. Cultural Competence (e.g. Cross cultural care, culturally safe and appropriate practice)
21. Therapeutic Nursing Behaviours/Respectful of personal space
22. Efficient and Effective Communication (e.g with professionals in other disciplines)
23. Communication and Documentation i.e. Verbal including handovers and non-verbal including documentation
24. Learner/Evidence Based Practitioner (e.g. Appropriate application of practice evidence)
25. Critical Analysis & Reflective Thinking (e.g. Use of Reflection and critical incidents, evidence of linking theory to practice)
26. Demonstrates Teaching/Educator skills (e.g. Utilising appropriate teaching & learning strategies in practice)
27. Acts as a Resource
28. Promotes self-care (e.g. specific gender and lifespan related information and strategies)
29. Demonstrates behaviour conducive to learning (e.g. approachable and supportive)
30. Learning and developmental culture - Learning environment (e.g. Relates to an environment conducive to learning and personal and professional growth as a new graduate)
