# Supplementary material for: What level of competency do experienced nurses expect from a newly graduated registered nurse? Results of an Australian modified Delphi study
Source: BMC Nurs. 2016 Jul 22;15:45. doi: 10.1186/s12912-016-0166-2 (PMC4957913; doi:10.1186/s12912-016-0166-2)
Supplement: Additional file 2: — Literature Search Strategy. (DOCX 39 kb) [file 12912_2016_166_MOESM2_ESM.docx]

Additional file 2: Literature Search Strategy

A literature search and review to locate research on competency levels for newly graduated RNs explored: ProQuest Central, Scopus, Medline, ERIC, Web of Science, Academic Search Complete, Australian Digital Thesis, Informit databases, and Science Direct. The search included: English language; from 1980 to 2013; peer reviewed journals, texts and theses. Search terms used were “competenc*”, “nurs*”, “registered nurse”, “newly graduated nurse*” and a variation of truncations. There were over 3000 papers identified from the above terms however much of this literature related to a number of particular areas. Firstly the competence of the registered nurse (not specifically new graduates) in acute care (hospital based settings such as medical, surgical or intensive care) and how that competence is maintained over time with just one study exploring Mental health nursing. Secondly how to assess ‘generic’ competence rather than what the skills that a newly graduated registered nurse should be competent in. Moving on to assessment strategies including how self-assessment or self-reporting of competence might be undertaken; then the impacts of ‘residency programmes’ or forms of ‘preceptorship’ on the developing (usually self-reported) competence. These were not relevant to this study. It is interesting to note that there were no studies exploring the skills or competency set of the new comprehensively prepared graduate nurse in Australia – interesting as this is an area often anecdotally stated by employers that new graduates are ‘not practice ready’.
