# Supplementary material for: What level of competency do experienced nurses expect from a newly graduated registered nurse? Results of an Australian modified Delphi study
Source: BMC Nurs. 2016 Jul 22;15:45. doi: 10.1186/s12912-016-0166-2 (PMC4957913; doi:10.1186/s12912-016-0166-2)
Supplement: Additional file 3: — Participant demographics. (DOCX 40 kb) [file 12912_2016_166_MOESM3_ESM.docx]

Additional file 3: Participant demographics

| Demographics characteristics | n = 299 | % |
| --- | --- | --- |
| Primary Role  Nursing academic  Clinical nurse consultant/manager/specialist  Clinical/Nurse Educator  Senior Nurse Manager  Other | 80  73  64  28  49  n=294 | 27.3  24.8  21.8  9.5  16.6 |
| Time in current role  < 1 year  1 – 2 years  3 – 5 years  6 – 10 years  > 11 years | 31  36  67  35  71  n=240 | 13.0  15.0  27.8  14.6  29.6 |
| Area of Practice  Acute Care  Aged Care  Community/Primary Care  Mental health  Multiple areas of practice | 61  30  14  43  91  (n=249) | 25.6  12.6  5.6  18.0  38.2 |
