# Supplementary material for: What level of competency do experienced nurses expect from a newly graduated registered nurse? Results of an Australian modified Delphi study
Source: BMC Nurs. 2016 Jul 22;15:45. doi: 10.1186/s12912-016-0166-2 (PMC4957913; doi:10.1186/s12912-016-0166-2)
Supplement: Additional file 4: — Top and bottom six skills areas ranked presented by respondent role. (DOCX 41 kb) [file 12912_2016_166_MOESM4_ESM.docx]

| Top and bottom six skills areas ranked presented by respondent role | | | | | |
| --- | --- | --- | --- | --- | --- |
|  | **RANK** | **(Ranking** based on mean scores) | | | |
|  |  | **All respondents** | **Acute Care** | **Academics** | **Mental Health** |
| 🡻TOP SIX🡻 | 1 | Privacy and Dignity (4.81) | Privacy and Dignity (4.75) | Privacy and Dignity (4.77) | Privacy and Dignity (4.81) |
|  | 2 | Demonstrates behaviour conducive to learning (4.69) | Demonstrates behaviour conducive to learning (4.74) | Demonstrates behaviour conducive to learning (4.71) | Demonstrates behaviour conducive to learning (4.73) |
|  | 3 | Personal Care – Provision and coordination (4.62) | Efficient and effective communication (4.58) | Personal Care – Provision and coordination (4.71) | Efficient and effective communication (4.63) |
|  | 4 | Efficient and effective communication (4.57) | Personal Care – Provision and coordination (4.56) | Efficient and effective communication (4.62) | Professional Nursing Behaviours - includes collaborative approaches to care (4.64) |
|  | 5 | Communication and documentation (4.52) | Communication and documentation (4.56) | Communication and documentation (4.57) | Personal Care – Provision and coordination (4.52) |
|  | 6 | Preventing Risk and Promoting Safety (4.51) | Planning of Nursing Care (4.52) | Preventing Risk and Promoting Safety (4.57) | Therapeutic nursing behaviour (4.47) |
|  | | | | | |
| 🡻 BOTTOM SIX 🡻 | 25 | Acts as a Resource (3.84) | Mental Health Nursing (3.77) | Mental Health Nursing (3.96) | Dealing with emotional and bereaved people. Includes conflict management (4) |
|  | 26 | Mental Health Nursing (3.83) | Dealing with emotional and bereaved people. Includes conflict management (3.75) | Demonstrates Teaching/Educator skills (e.g. Utilising appropriate teaching & learning strategies in practice) (3.94) | Demonstrates Teaching/Educator skills (e.g. Utilising appropriate teaching & learning strategies in practice) (3.9) |
|  | 27 | Demonstrates Teaching/Educator skills (e.g. Utilising appropriate teaching & learning strategies in practice) (3.81) | Demonstrates Teaching/Educator skills (e.g. Utilising appropriate teaching & learning strategies in practice) (3.69) | Dealing with emotional and bereaved people. (3.9) | Acts as a Resource (3.85) |
|  | 28 | Supervisory Skills (3.68) | Supervisory Skills (3.64) | Leadership Skills (3.64) | Supervisory skills (3.76) |
|  | 29 | Leadership Skills (3.62) | Leadership Skills (3.61) | Supervisory Skills (3.63) | Leadership Skills (3.71) |
|  | 30 | Case manager (3.43) | Case manager (3.37) | Case manager (3.4) | Case Manager (3.69) |

Additional file 4: Top and bottom six skills areas ranked presented by respondent role.
